# 

**Published:** 1881-10

**Authors:** 


### Pamphlets.

Transactions of the Mississippi State Medical Association, 1881. B. F. Ward, M.D., Winona, President; Wirt Johnson, M.D., Jackson, Secretary.

First Annual Report of the Astronomer in Charge of the Horological and Thermometric Bureaus of the Winchester Observatory of Yale College. 1880–81. By Leonard Waldo.

A Probable Cause of Tardy, Painful Labor, not Hitherto Recognized. By George H. Rohe, M.D., Baltimore. Reprint from the *New York Medical Journal and Obstetrical Review.*

Abortive Treatment of Pneumonia. By W. Y. Gadbury, M.D., Yazoo City. Reprint from Transactions of the Mississippi State Medical Association.

